# Estimating False Positive Contamination in Crater Annotations from Citizen Science Data

**DOI:** 10.1007/s11038-016-9499-9

**Published:** 2016-11-19

**Authors:** P. D. Tar, R. Bugiolacchi, N. A. Thacker, J. D. Gilmour, MoonZoo Team

**Affiliations:** 1 Centre for Imaging Sciences Stopford Building, University of Manchester, Oxford Road, Manchester, M13 9PT UK; 2 School of Earth, Atmospheric and Environmental Sciences Williamson Building, University of Manchester, Manchester, M13 9PL UK; 3 Space Science Institute, Macau University of Science and Technology, Macau, China

**Keywords:** Linear Poisson Models, Moon Zoo, Citizen science, 62P35, 65G99

## Abstract

Web-based citizen science often involves the classification of image features by large numbers of minimally trained volunteers, such as the identification of lunar impact craters under the Moon Zoo project. Whilst such approaches facilitate the analysis of large image data sets, the inexperience of users and ambiguity in image content can lead to contamination from false positive identifications. We give an approach, using Linear Poisson Models and image template matching, that can quantify levels of false positive contamination in citizen science Moon Zoo crater annotations. Linear Poisson Models are a form of machine learning which supports predictive error modelling and goodness-of-fits, unlike most alternative machine learning methods. The proposed supervised learning system can reduce the variability in crater counts whilst providing predictive error assessments of estimated quantities of remaining true verses false annotations. In an area of research influenced by human subjectivity, the proposed method provides a level of objectivity through the utilisation of image evidence, guided by candidate crater identifications.

## Introduction

Moon Zoo (Joy et al. 2011) was a citizen science project aiming to catalog small lunar craters by allowing volunteers to annotate candidates using a web-based interface between 2010 and 2015. The Moon Zoo custom Graphical User Interface (GUI) was an Adobe Flash application based on the ActionScript programming language. The software Application Programming Interface and database layer were developed by the Zooniverse team at Oxford University, building on their experience with storing and analysing large amounts of citizen science data (Lintott et al. 2008). Moon Zoo was similar in design and aim to alternative projects including MoonMappers (Robbins et al. 2014). One major goal of the Moon Zoo project was to gather crater statistics for the plotting of crater Size Frequency Distributions (SFDs). However, the raw annotations contain significant contamination from misidentified craters which must be corrected for if crater counts are not to be biased.

SFDs are commonly used for investigating the evolution of planetary surfaces. A conventional SFD plots the cumulative frequencies of craters falling into geometrically increasing size-bands, normalised to a unit of surface area, thereby describing geological units in terms of crater diameters and densities (Neukum et al. 1975). On a single body, differences among SFDs allow a relative sequence of events to be established. This requires an appropriate treatment of uncertainty to answer questions such as whether two features are consistent with a single formation event or multiple events. SFDs estimated from lunar regions for which radiometrically-dated samples are available can be used to calibrate Chronology Functions which might be applicable for dating other regions or even other planetary surfaces (Neukum et al. 2001).

The quantitative use of crater statistics to establish ages must incorporate a good understanding of the uncertainties in estimated crater counts. Conventional crater counting assumes Poisson errors (i.e. $\sqrt{N}$) on crater counts (Arvidson et al. 1979). These errors are propagated to give errors on surface age estimates. The underlying Poisson assumption has become part of standard SFD analysis software (Michael and Neukum 2010). However, repeatability studies of experts and community crater counters (Robbins et al. 2014) reveal uncertainties in counts far larger than those arising from Poisson perturbations alone. Earlier work has also shown subjective sources of uncertainty in both crater counts (Greely and Gault 1970; Kirchoff et al. 2011) and size estimates (Gault 1970). Empirical error rates used to assess automated crater detectors also reveal systematic effects which violate the simple Poisson assumption, i.e. false positive and negative detection, for examples see Kamarudin et al. (2012), Kim et al. (2005) and Bandeira et al. (2007).

**Fig. 1:**
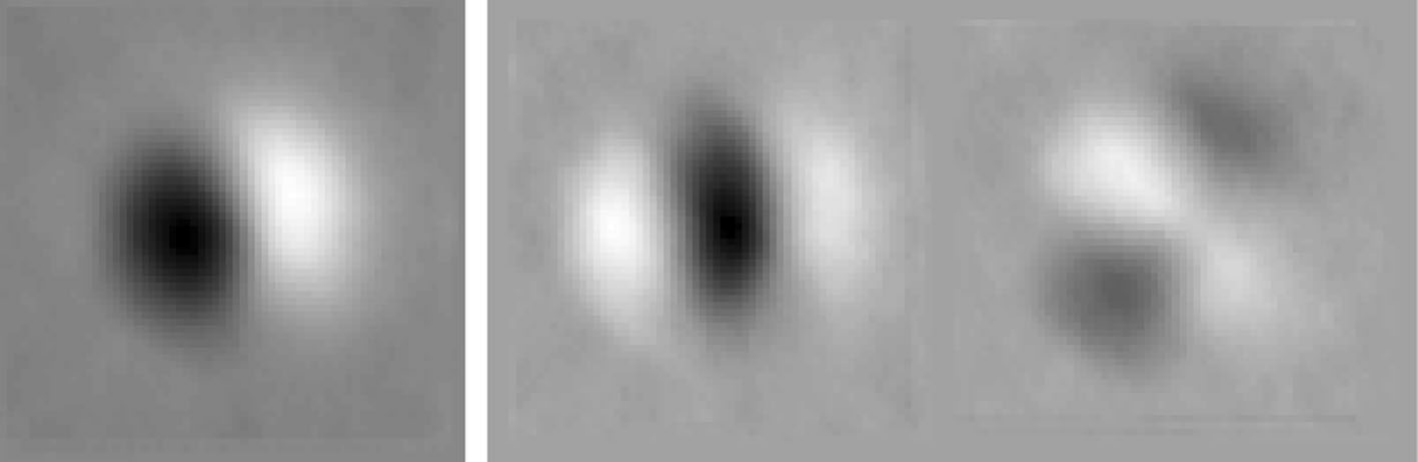
*Left* mean grey level crater template derived from Moon Zoo data. *Right* Combined horizontal and vertical gradient (x, y derivative) template

The Moon Zoo crater data Bugiolacchi et al. (2016) is known to contain numerous false positives, where ambiguous features (shadows, topographic highs etc.) have been erroneously annotated. Many craters have also been annotated using a default size setting inappropriate for many sized craters. In addition to false positives which artificially boost crater counts, false negatives also occur where real craters are not annotated. This present work focuses on addressing false positive contamination, i.e. features which have been annotated, but are not genuine craters. A simple approach to identifying false positives is to apply a threshold on the number of users who have annotated a particular crater. This assumes that multiple people are less likely to make the same misidentification (Robbins et al. 2014). However, this approach requires a greater number of annotations to catch all craters, risking discarding real craters which have only been marked a small number of times. Alternatively, even in places with few annotations, a pattern recognition system could be trained to probabilistically weight annotations using image evidence and training data (Fig. 1). This work shows how Linear Poisson Models (LPM), a supervised learning system based upon Likelihood regression (Tar and Thacker 2014; Tar et al. 2015), can be applied to crater annotations to reduce uncertainty in contaminated counts. Unlike alternative machine learning methods such as Support Vector Machines (Steinwart and Christmann 2008) and Random Forests (Ho 1995), LPMs incorporate an error theory for predicting the stability of estimated quantities of identified true and false features.

This work does not intend to present any lunar science results or generate conventional SFDs. Rather, it is intended to show how one particular problem, that of false positives, can be reduced in raw crater annotations. The method is demonstrated using Moon Zoo data with bootstrap resampling in order to show that:

- Linear Poisson Model regression can estimate false positive contamination levels in Moon Zoo data;
- the estimation error on the corrected counts can be predicted using the LPM’s error theory, and the observed error in practice matches the predictions;
- that self-consistent results can be achieved over a range of different crater quantities and using different image evidence.

A more comprehensive correction, also taking into consideration missing craters from false negatives, would be required to fully correct counts suitable for SFDs. This additional correction step is outside the scope of the current work.

## Methodology

Given a database of crater annotations known to contain contamination, the problem we address is that of assigning probabilities to each annotation, permitting correctly weighted total counts to be achieved. A solution must also provide an honest indication of the uncertainty remaining in the corrected counts, i.e. error bars, which should have predictive value.

**Fig. 2:**
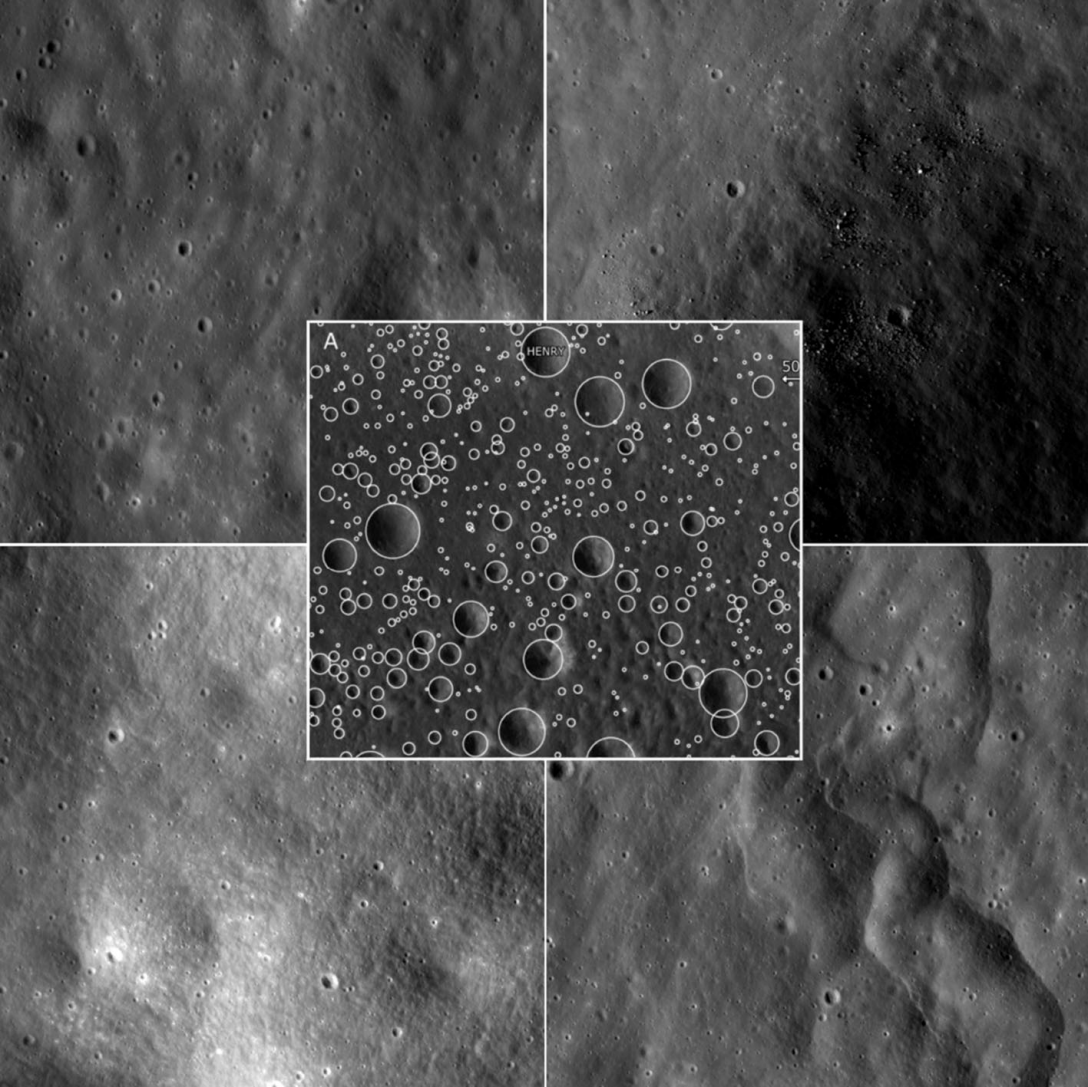
Examples of terrain found within NAC images M104311715LE/RE. The Moon Zoo dataset contains uniform homogeneous regions, as well as *dark* areas with significant boulders. The *central panel* shows examples of Moon Zoo candidate crater annotations

The false positive correction process begins with a set of candidate crater annotations and a high resolution lunar image. The annotations used in this work were taken from a subset of Apollo 17 craters (NAC M104311715LE and M104311715RE) pre-processed using the clustering described in Bugiolacchi et al. (2016). 20,000+ annotations were used, each consisting of x and y centre coordinate parameters, a diameter and a parameter for the number of individuals who highlighted that particular candidate crater. No user annotation threshold was used to pre-filter the annotations, therefore all candidates within the subset were used, including those annotated by only single users. Figure 2 provides examples of the terrain found within the images, as well as a sample of Moon Zoo annotations. Briefly, the process is applied and tested using the following steps:

- A subset of ground truth must be agreed upon containing examples of “true” and “false” craters to be used as training and testing data. For testing purposes here, the 20,000+ crater annotations were visually inspected by the first author, 25% of which were deemed false positive (Sect. 2.1);
- A template image of a crater is created using the average computed appearance from a set of “true” craters. Two template types are tested, one making use of pixel intensity and another using horizontal and vertical pixel derivatives (Sect. 2.2);
- A similarity measure is constructed for comparing crater templates to individual annotations giving a match score to measure the strength of image evidence supporting there being a real crater at each candidate location (Sect. 2.2.1);
- The distribution of template match scores across image regions is sampled for “true” and “false” craters, resulting in histograms of template responses to different classes of annotation (Sect. 2.2.2);
- The histograms of template responses are used to train a Linear Poisson Model (LPM), which results in a set of Probability Mass Functions (PMFs) which can be combined linearly to describe the distribution of matches in future data (Sect. 2.2.3);
- The LPM is fitted using an Expectation Maximisation algorithm to candidate crater match scores that require correction. This results in estimated quantities of “true” and “false” classes of annotations (Sect. 2.2.3);
- The LPM’s error theory is applied to predict the error bars on the estimated “true” and “false” crater quantities (Sect. 2.2.4). These predictions are then tested by repeatedly resampling and analysing annotations to compare predicted distributions to actual spreads of counts.

These steps are describe in detail below before testing using bootstrap resampling from Moon Zoo crater annotations.

### Ground Truth

The method presented here can be thought of as objectively fitting a subjective definition of craters’ appearances. For the method to operate a ground truth training data set is required which can be defined by an expert or via consensus. As the method is designed to be objective in its application, the minimum requirement is that the training and testing sets make use of the same definition of true and false craters. To ensure this condition, the ground truth defined in this work was used to both train and test, with independent subsets being generated using bootstrap re-sampling. Given the lack of any absolute and objective ground truth, we argue that this level of consistency is the best that can reasonably be achieved.

Approximately 20,000 candidate craters from Moon Zoo with diameters of 20 pixels or greater were individually assessed and categorised into “true” and “false” examples by the first author. Greater than 99% of these were small independent craters, i.e. non-intersecting. Approximately $\frac{1}{4}$ of the craters were deemed to be “false”. These were gathered from the following NAC images: M104311715LE and M104311715RE, which are approximately 5,000 by 50,000 pixels in size.

It might be argued that training and testing would be better performed within different size-bands of craters to cater for morphological differences at different scales. However, as a proof-of-concept and to maintain a large number of test subjects, all sized craters within the Moon Zoo database were used jointly. The largest craters, of which there are very few, are in the region of 100 pixels in diameter (approx. 200 m). Much larger craters with distinctive morphologies (e.g. central rings and significant impacts within impacts) were never part of the database.

### Crater Template Construction

LPMs model data densities as linear combinations of histograms, with parameters estimated via Likelihood. They assume data can be described as an additive mix of independent Poisson variables. As LPMs work with histogram data, the image evidence around candidate craters must be encoded somehow into appropriately binned frequencies. Image locations containing candidate craters can be compared to template crater images to give a measure of how crater-like the candidates actually are. Histograms of the level of template match can be populated for anaysis.

Two different forms of template are investigated: an average crater appearance which has had the local mean pixel grey level subtracted; and a derivative template modelling changes in illumination both horizontally and vertically across an average crater. Examples of these templates can be seen in Fig. 1. Both types of template begin with multiple crater image examples scaled to a common size. For each example, the mean grey level over the area of the image is subtracted from each pixel. This helps to normalise the images to take into account local reflectance artifacts. The average template is given by the mean per-pixel values computed across all examples. Two derivative subtemplates are constructed, one from the mean pixel differences between adjacent pixels to the left and right, and one from the mean differences above and below; the horizontal and vertical subtemplates are then concatenated next to one another. The derivative template further reduces the effects of mean local grey levels and albedo variations by focusing on differences rather than absolute values (Fig. 1, right).

The grey level templates used in the experiments were 60 by 60 pixels in size, whereas the composite derivative templates were 120 by 60. The craters were scaled to fit within the templates with a 40 pixel diameter and 10 pixel border. All craters during testing show similar illumination direction and inclination. Whilst a more elaborate template construction method may improve generalisation to other illumination conditions (such as appearance modelling or shape from shading), the relatively simple templates adopted here maintain easy to understand noise characteristics making a similarity measure easier to construct.

#### Similarity Measures

LPMs are designed to be quantitatively self-consistent so that conclusions drawn from an analysis should be invariant to how measurements are taken. This is in-line with standard scientific requirements that choices in setup should not lead to different interpretations. However, different measurement choices can lead to higher or lower accuracy, i.e. larger or smaller error bars on final summaries. To illustrate this, two different forms of similarity measure are investigated: the mean of squared residuals between image and template pixels (i.e. mean squared error, $S_{MSE}$); and a normalised dot-product which treats image and template pixels as vectors ($S_{DP}$). Both of these measures are motivated by a Likelihood interpretation of template matching which assumes independent Gaussian noise on image pixels:

1 $$\begin{aligned} {\mathcal {L}}_{match} = \prod _i^n e^{-(a_i-b_i)^2} \end{aligned}$$

where *a* is the template; *b* is the image patch being matched; *i* is an index over each pixel; and *n* is the number of pixels in the template. Note that as this function (or rather those related to it below) is searched for maxima, the absolute normalisation is unimportant. It can be seen that maximising this Likelihood is equivalent to minimising $S_{MSE}$:

2 $$\begin{aligned} S_{MSE}= & {} \frac{1}{n} \sum _i^n (a_i-b_i)^2 \end{aligned}$$

3 $$\begin{aligned} \ln {\mathcal {L}}_{match}= & {} -\sum _i^n (a_i-b_i)^2 \end{aligned}$$

where the peak in the log Likelihood coincides with the peak in the Likelihood and $S_{MSE} \propto - \ln {\mathcal {L}}_{match}$. It can also be seen that maximising $S_{DP}$ is approximately equivalent to minimising $S_{MSE}$ by inspecting:

4 $$\begin{aligned} S_{DP}= & {} \frac{1}{n \Vert a\Vert } \sum _i^n a_i b_i \end{aligned}$$

5 $$\begin{aligned} S_{MSE}= & {} \frac{1}{n} \left( \sum _i^n a_i^2 + \sum _i^n b_i^2 - 2\sum _i^n a_i b_i \right) \end{aligned}$$

where $\sum _i^n a_i^2$ and $\Vert a\Vert$ (length of the vector defined by the pixel values of the template) are constant if a single fixed size template is used; and $\sum _i^n b_i^2$ is dominated by the mean grey level of the image patch being matched, which is approximately constant within a local image region. Dividing by The $S_{DP}$ similarity measure therefore focuses on matching the high spatial frequency components of the crater templates which provides further invariance to local illumination and albedo effects.

#### Applying the Templates

The construction of templates and selection of similarity measures presented above assume the only sources of variability are local illumination conditions and pixel-level noise. However, the degradation state of craters also significantly affects appearance. To approximately accommodate the effects of degradation, images being matched are first smoothed using Gaussian blurring, as the blurring of a crater image visually mimics the effects of erosion, allowing for improved matches. This modelling need not be perfect (the simulated degradation may be very approximate), as the subsequent supervised learning stage using LPMs will model response variations including small changes in morphology and degradation. The match score assigned to a given candidate crater is computed as follows:

1. smooth the crater image by a small amount;
2. subtract the mean local grey level;
3. compute horizontal and vertical derivatives if the derivative template is to be used;
4. compare the template to the crater using one of the similarity measures;
5. repeat the process for different smoothing levels until the best match score is achieved;

To achieve best template matches, 16 logarithmic levels of image smoothing were employed, which are listed in Table 1.

**Table 1:** Table of Gaussian smoothing filter widths in pixels

1	2	3	4	5	6	7	8
0.10	0.12	0.14	0.17	0.21	0.25	0.30	0.36
0.43	0.52	0.62	0.74	0.89	1.07	1.28	1.54

As noted earlier, a LPM analysis should provide consistent conclusions irrespective of the exact measurements used as input. Alternative inputs to the LPM analysis were in the form of one and two dimensional match score histograms. In the one dimensional cases, all the four possible combinations of templates and similarity measures used are listed in Table 2.

**Table 2:** Table of the four possible combinations of templates and similarity measures

	Avg. appearance (grey level)	Avg. derivative (gradient)
$S_{MSE}$	Grey MSE	Grad MSE
$S_{DP}$	Grey DP	Grad DP

Additionally, pairs of match scores were combined into joint histogram distributions with 2 dimensions giving the 6 combinations in the table below. These allowed information from the different templates and match scores to be analysed jointly with the aim of improving accuracy and checking consistency. These are listed in Table 3.

**Table 3:** Table of the four possible combinations of templates and similarity measures

Dimension 1	Dimension 2
Grad MSE	Grad DP
Grad MSE	Grey DP
Grey DP	Grad DP
Grey MSE	Grad DP
Grey MSE	Grad MSE
Grey MSE	Grey DP

These different histograms each contain subtly different information regarding the image evidence used to differentiate between true and false craters. By applying LPMs to each of these possible combinations we aim to: (a) demonstrate the consistency of the method by showing that equivalent results can be achieved for the different evidence used; (b) select the most efficient combination to achieve best absolute levels of counting repeatability.

#### Training and Fitting LPM

A LPM (Tar and Thacker 2014; Tar et al. 2015) can describe the shape and variability of distributions found within histograms using a linear combination of simpler fixed components^1^. In the case of crater match score histograms, one set of components describes “true craters” and one set describes “false craters”. To the extent that the histograms are different we can distinguish true craters from false craters using LPMs:

6 $$\begin{aligned} {\mathbf {H}} \approx {\mathbf {M}} = {\mathbf {P}} {\mathbf {Q}} \end{aligned}$$

where ${\mathbf {H}}$ is a histogram of match scores under analysis, with bins ${\mathbf {H}}_X$ (*X* being an interval defining a range of match scores); ${\mathbf {M}}$ is the model; ${\mathbf {P}}$ is an *m* by *n* matrix describing the Probability Mass Functions (PMFs) of *n* components with elements ${\mathbf {P}}_{ij} = P(X=i|k=j)$, i.e. the probability of an entry in bin *X* (i.e. a match score range) given component *k* (which belongs to either a “true” or “false” crater class); and ${\mathbf {Q}}$ is a column vector of *n* quantities corresponding to the amount of each component present within the histogram.

During training, a LPM must determine the necessary PMFs required to describe the distribution of “true” and “false” crater match scores. Once these have been established they can be fitted to new histograms containing unknown quantities of contamination, thus estimating how much of each category exists within the data. Both training and fitting are achieved using Expectation Maximisation (Dempster et al. 1977) to optimise the following Extended Maximum Likelihood (Barlow 1989):

7 $$\begin{aligned} \ln {\mathcal {L}} = \sum _X \ln \left[ \sum _k P(X|k){\mathbf {Q}}_k\right] {\mathbf {H}}_X - \sum _k {\mathbf {Q}}_k \end{aligned}$$

During training, this function is jointly optimised for a set of example histograms giving a set of *P*(*X*|*k*) components. This is performed separately for the “true” and “false” classes, resulting in a set of PMFs associated with each class. The number of components required to describe each class is determined by adding additional components until the $\chi ^2$ per degree of freedom between LPM and example histograms approaches unity. During contamination estimation in new data, this function is optimised to fit true/false classes of component by adjusting weighting quantities only, ${\mathbf {Q}}_k$, which are then summed within their respective classes to give total quantities of “true” craters and “false” contamination.

The adequacy of the LPM to describe the match score histograms is quantitatively testable, unlike many alternative machine learning methods. The $\chi ^2$ per degree of freedom function for assessing the quality of a fitted histogram distribution is defined as:

8 $$\begin{aligned} \chi ^2_{(m-n)} = \frac{1}{m-n} \sum _X \frac{\sqrt{\sum _k P(X|k){\mathbf {Q}}_k} - \sqrt{{\mathbf {H}}}_X }{\frac{1}{4}} \end{aligned}$$

where *m* is the number of histogram bins and *n* is the number of components estimated. The square roots stabilise the Poisson bin frequencies (Anscombe 1948), giving them a constant variance of $\frac{1}{4}$.

#### Quantity Error Estimation

Sampling errors in training histograms and incoming data combine to give a level of uncertainty on the estimated quantities. In order to factor these uncertainties into final crater counts they must be propagated through the EM algorithm using error propagation (Barlow 1989):

9 $$\begin{aligned} {\mathbf {C}}_{Q}& = {} {\mathbf {C}}_{data} + {\mathbf {C}}_{model} \end{aligned}$$

10 $$\begin{aligned} {\mathbf {C}}_{ij(data)}= & {} \sum _X \left[ \left( \frac{\partial {\mathbf {Q}}_i}{\partial {\mathbf {H}}_{X}} \right) \left( \frac{\partial {\mathbf {Q}}_j}{\partial {\mathbf {H}}_{X}} \right) \sigma ^2_{{\mathbf {H}}_{X}} \right] \end{aligned}$$

11 $$\begin{aligned} {\mathbf {C}}_{ij(model)}= & {} \sum _X \left[ \sum _k \left( \frac{\partial {\mathbf {Q}}_i}{\partial {\mathbf {H}}_{X|k}} \right) \left( \frac{\partial {\mathbf {Q}}_j}{\partial {\mathbf {H}}_{X|k}} \right) \sigma ^2_{{\mathbf {H}}_{X|k}} \right] \end{aligned}$$

where ${\mathbf {C}}_{Q}$ is the error covariance matrix for the estimated quantities; ${\mathbf {C}}_{data}$ is the statistical contribution of the error from the incoming histogram data; and ${\mathbf {C}}_{model}$ is the systematic contribution from the training exemplar histograms used to construct the LPM. The relative contribution from both sources of error change as a function of the ratio of training to testing data. These covariance estimates form the basis of SFD error bars.

#### Interpreting Quantities

For a given input cohort of candidate craters, two key numbers emerge from the LPM analysis: an estimate of the number of false positive craters in the cohort and an estimate of the number of true craters. It should be noted that the Likelihood regression technique does not at any point make decisive labelling decisions regarding individual candidate craters, i.e. a list of specifically identified false craters and their locations are not an output. Instead, the output quantities are computed by integrating over probabilities. For instance, if there are 100 craters, all deemed to have a 90% probability of being ‘true’, each candidate contributes 0.9 to the count of true positives, yielding a total of 90 craters. This total acknowledges that there are likely to be 10 false positives in the set, but cannot say exactly which ones are false. What we consider important for science is that for a given cohort (e.g. a particular geological unit) the number of craters estimated does not deviate by more than the error predicted by LPMs. This criteria motivates a testing strategy involving large numbers of repeated counts, compared against defined ground truth to check error distributions, not just spot values.

Regarding the objectivity of results, any output quantities can only be as good as the training data provided. If a training set contained a biased distribution of craters then output quantities would also be biased. The output quantities report number of craters consistent with the provided training data only. This condition is also reflected within the testing strategy, which repeatedly draws cohorts of craters from a common pool of predefined candidates. In this way, errors show deviation from definitions, which should be no larger than predicted by the LPM error theory.

## Experiments

For the method to be deemed successful, the primary criterion is that the statistical distribution of estimated corrected quantities is predicted by their theoretical error distributions, i.e. the quantities, ${\mathbf {Q}}_k$, are distributed around the true values with covariances given by ${\mathbf {C}}_Q$. Put more simply, over repeated trials the difference between corrected crater counts and defined ground truth should be no more (or indeed no less) than the spread of values predicted using the LPM error theory. This criterion provides a test of the LPM theory, the appropriateness of its application, and also the implementation of software.

In order to assess this, it is necessary to make repeated measurements which can be compared to predefined ground truth values. To facilitate this, LPMs (Sect. 2.2.3) were repeatedly constructed and applied to estimate the quantity of “true” and “false” craters in randomised samples, with 1000 repeated measurements made in order to produce predicted and empirical error distributions on the estimated quantities. During each trial craters were selected from random rectangular regions of the NAC images, sampled with replacement. Regions rather than individual craters were selected to preserve any local spatial correlations. Sampling with replacement (Barlow 1989), where selected regions could overlap or be used multiple times, was used to achieve the required quantity of data for thorough testing. To ensure that each drawn cohort was statistically unique and as independent as possible, a small quantity of image noise was artificially added to each region, and regions were permitted to be combined to help randomise the cohort. Whilst this bootstrap resampling method applies several techniques to randomise each test cohort, it is acknowledged that the underlying data source is a fixed finite pool and therefore a possible source of correlation. However, we believe that any systematic correlations caused by the common data pool should become a systematic part of the linear model PMFs, which are already subject to systematic biases due to choice of training data.

The different contributions to the error, ${\mathbf {C}}_{data}$ and ${\mathbf {C}}_{model}$, are a function of the training to testing ratio. A range of relative data quantities were tested, using 0.01, 0.10, 1.00, 10.00 and 100.00 times as much testing data as training data. The quantity of contamination within each trial matched that found within the raw MoonZoo data, which was approximately one quarter, as determined by the manual inspection which defined the reference ground truth.

After each trial the difference between known ground-truth values and estimated values were divided by the predicted error and recorded. If successful, this should give a distribution of recorded values with a mean of zero and standard deviation of unity. The predicted accuracies were also recorded as percentage errors on measured quantities to assess absolute levels of accuracy attainable.

**Fig. 3:**
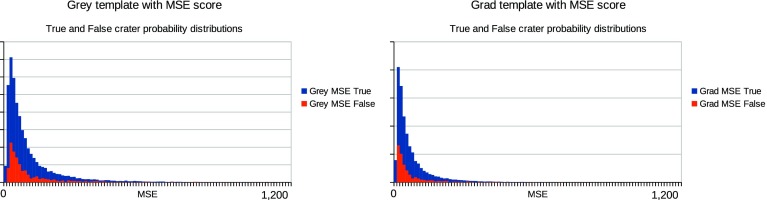
*Left* mean squared error match score distribution computed using grey level image template. *Right* MSE match score distribution computed using gradient image template

**Fig. 4:**
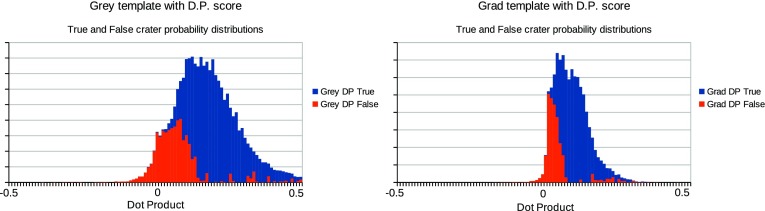
*Left* dot product match score distribution computed using grey level image template. *Right* DP match score distribution computed using gradient image template

**Fig. 5:**
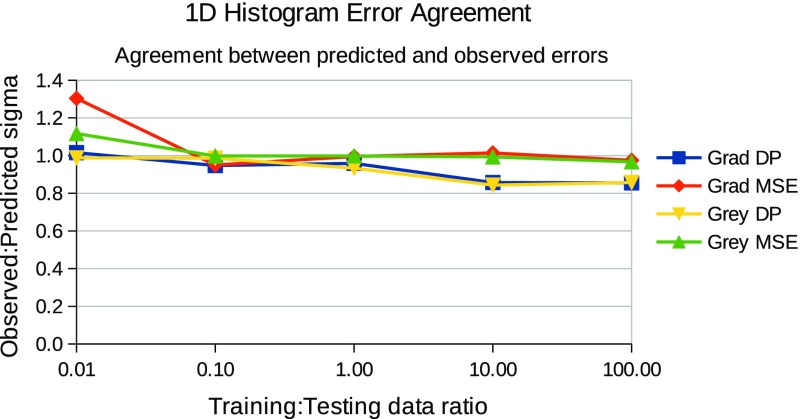
Corroboration that predicted measurement errors are seen in practice when linear models are constructed and fitted using 1D match score histograms. The x-axis indicates the relative quantities of training and testing data. The y-axis shows observed errors over 1000 trials per point divided by the predicted errors

In summary, for each choice of template and match score combination the testing followed the steps below:

- For each relative quantity of training and testing data, repeat the following steps
- Gather a known quantity of “true” and “false” craters from the defined ground truth, fit templates and record histograms of match scores (example histograms can be seen in Figs. 3 and 4.
- Train a LPM to recognise the classes of crater (true and false)
- Gather an independent known quantity of “true” and “false” craters from the available data by sampling with replacement and adding a small quantity of noise to the crater images, again to increase statistical independence of the samples
- Record histograms of match scores again
- Regress LPM to new histograms and estimate quantities of “true” and “false” craters
- Compare quantities to known ground truth, dividing the difference by the error predicted by the LPM. The result, which should be unity if estimates are within errors can be seen in Figs. 5 and 7.
- Repeat 1000 times per quantity and template/match score combination.

## Discussion

The method assumes that there are significant differences between the distribution of match scores for “true” and “false’ craters, which provides information allowing features to be distinguished. The distribution of match scores for the four combinations of template and similarity measure can be seen in Figs. 3 and 4. The differently shaded regions correspond to match score values for false positives and “true” craters. In the case of the $S_{MSE}$ match score distributions (Fig. 3), the true and false positive distributions are subtly different on visual inspection, with the true craters having longer tails and larger modes. In contrast, the $S_{DP}$ distributions are clearly different between the true and false craters, suggesting that this match score should be better at differentiating between the two cases.

**Fig. 6:**
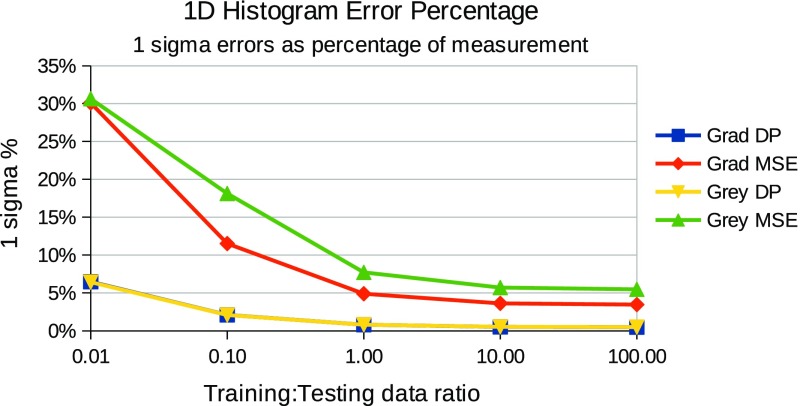
Measurement errors as percentage of measured quantities when using 1D match score histograms. The x-axis indicates the relative quantities of training and testing data. The y-axis shows one standard deviation of predicted accuracies as a percentage of the measurement

**Fig. 7:**
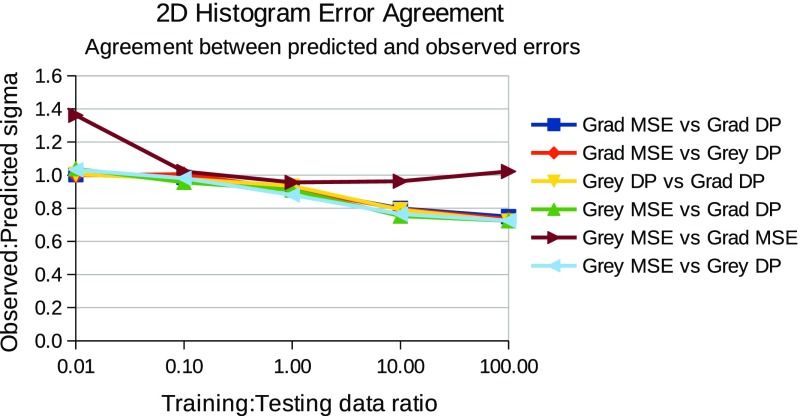
Corroboration that predicted measurement errors are seen in practice when linear models are constructed and fitted using 2D match score histograms. The x-axis indicates the relative quantities of training and testing data. The y-axis shows observed errors over 1000 trials per point divided by the predicted errors

**Fig. 8:**
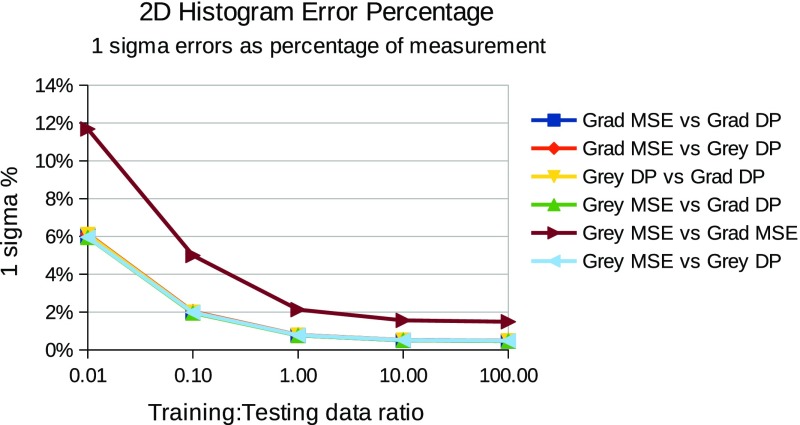
Measurement errors as percentage of measured quantities when using 2D match score histograms. The x-axis indicates the relative quantities of training and testing data. The y-axis shows one standard deviation of predicted accuracies as a percentage of the measurement

Evidence of successfully estimated quantities of “true” craters verses contamination can be seen in Figs. 5 and 7, for individual ($S_{MSE}$ and $S_{DP}$ separately) and joint ($S_{MSE}$ and $S_{DP}$ combined in 2 dimensions) match score distributions respectively. These show that estimated quantities match ground truth quantities, within predicted errors, i.e. that the predicted accuracies match observed accuracies over repeated trials. The x-axis shows the ratio of training to testing data, and y-axis shows the average ratio of observed to predicted errors, as predicted from Eq. 9 and empirically measured over 1000 trials. The general trend shows a ratio of unity across the majority of the plots, showing that both the statistical and systematic contributions to the errors (which are a function of training and testing quantities) are correctly estimated using Eqs. 10 and 11. The instabilities seen in the joint plots, where there is deviation away from a flat line at unity, can be explained by underpopulated histogram bins on the left (small data quantities) and growing model discrepancies on the right (large data quantities). However, in all cases the errors were predicted within a half of the actual errors. This can remove much of the danger of over-interpretation possible if conventional Poisson errors alone were assumed on counted craters. The actual accuracies attained, as percentages of estimated quantities, can be seen in Figs. 6 and 8, showing that accuracies improve as the quantity of data analysed increases. The best overall performance is achieved when the $S_{DP}$ similarity measure is used, which as noted above has the most distinctive distributions of “true” and “false” craters.

The use of LPMs has been shown to be statistically self-consistent, as quantities were successfully estimated to within predicted levels of accuracy under all tested conditions. Corrected counts were achieved for different types of template, similarity measures and quantities of data. The only significant differences between scenarios is seen in the size of counting errors, with $S_{MSE}$ scores at low quantities of data performing the worst and $S_{DP}$ scores at high quantities performing the best. This can be accounted for through two mechanisms: firstly, the normalised dot product provides greater separability of “true” and “false” craters, as their distributions overlap less than in the $S_{MSE}$ alternative (Figs. 3, 4); secondly, as the quantity of data increases, the errors improve through the availability of larger samples.

**Fig. 9:**
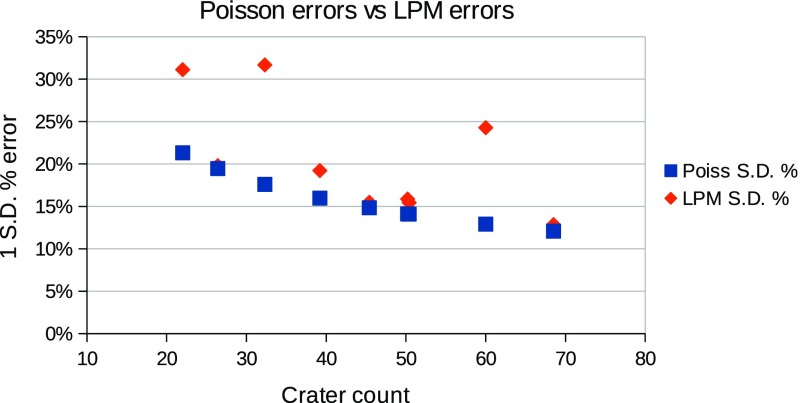
Poisson errors compared to predicted errors on false positive corrected crater counts from LPM covariances. The *squares* indicate 1 standard deviation percentage errors assuming conventional Poisson ($\sqrt{N}$) uncertainties. The *diamonds* indicate errors computed using the method in Sect. 2.2.4. On average, for these 8 counts the error on corrected counts is 1.3 times the Poisson error

The selection of templates and match scores used are not the only ones available, and no claim is being made that the most successful combination tested is the absolute best possible. However, the best total errors achieved are close to Poisson, as seen in Fig. 9. In counts that do not suffer from missing data, then the use of expertly trained LPMs could provide a mechanism for producing highly repeatable crater statistics, so long as a single “expert” definition could be agreed upon and adopted as a standard.

### Limitations

The simple crater templates used were only designed for small diameters and do not address problems associated with overlapping or nested craters. As such, the method would required better crater models if it were to be used in more complex and densely cratered terrains, such as the Lunar highlands. A range of different templates may also be required if the method is to be extended to a large range of crater sizes to account for changes in morphology associated with, for instance, the simple to complex transition.

The use of templates also limits the use of trained LPMs to images with the same solar illumination orientation, as changing shadows change the appearance of templates. It may be time consuming to train alternative LPMs for all required conditions. This should be seen as a semi-automated method, as each new dataset will likely require specific training where a subset of craters are re-examined by an expert in order to provide a representative training subset.

A further limitation caused by the method’s supervised learning nature is that of training data selection. Whilst the definition of an impact crater might be clear, i.e. ‘a morphologic feature produced as a result of a hypervelocity impact’, how this definition translates to judgments of a crater’s appearance is a source of subjectivity. As a semi-supervised system for individual use, the technique can be of value. However, for wide adoption, a large set of standards would be required for different terrains, morphologies and whole worlds. Given that fully automated crater counting systems are being researched by others, which also require training, there is a growing need to address the issue of reliable crater identification. This must include agreement between experts, whose variability in crater identification can itself be a significant source of uncertainty. This is a challenge for the wider crater counting community if appropriate tools are made available to them.

Finally, other problems associated with citizen science datasets have not been addressed, making this work a partial solution to correcting crater counts. In particular, missing data will result in negatively biased counts unless additional steps are taken.

## Conclusions

It is known that large variations exist between different experts and citizen scientists when counting craters. One option is to simply accept the levels of variability and plot larger error bars on crater counts. Another option is to try to build a consensus by taking mean counts, or accepting craters only if a sufficient number of individuals have identified them jointly. Alternatively, as shown in this work, corrections can be attempted which make objective use of image evidence, albeit using a subjective (but agreed upon) definition of craters. The Linear Poisson Model technique demonstrated has the advantage of providing theoretically predictive errors on corrected counts. In contrast, the other options (taking means or thresholding) still requires an empirical leap in order to understand the final count stabilities.

The key conclusions of this work are as follows:

1. Under our ground truth definition, MoonZoo crater counts contain approximately 25% contamination from false positives.
2. The effects of false positive contamination can be successfully quantified and corrected for using Linear Poisson Models, utilising objective differences in template crater match scores, so long as an appropriate standard template and ground truth can be established.
3. Statistically consistent results can be achieved for different types of templates and match scores.
4. The proposed methods are currently limited to small craters in sparsely cratered regions, but work could be undertaken to widen applicability.
5. The method is best suited to large datasets which are well populated (i.e. low false negatives) but suffer from contamination.

## Footnotes

1 This is a relatively new approach with few current examples. A method summary document, which has not been peer reviewed, is available as supplementary material for those wishing to implement this new LPM approach: http://www.tina-vision.net/docs/memos/2015-008.pdf.
